# Evaluation of emotional (depression) and behavioural (nutritional, physical activity and sleep) status of Turkish adults during the COVID-19 pandemic period

**DOI:** 10.1017/S136898002000498X

**Published:** 2020-12-09

**Authors:** Sevil Karahan Yılmaz, Günay Eskici

**Affiliations:** 1 Department of Nutrition and Dietetics, Faculty of Health Sciences, Erzincan Binali Yıldırım University, Erzincan, Turkey; 2 Çanakkale Onsekiz Mart University, Faculty of Sport Sciences, Department of Coaching Education, Canakkale 17100, Turkey

**Keywords:** COVID-19, Depression, Nutrition, Physical activity, Sleep

## Abstract

**Objective:**

To evaluate emotional (depression) and behavioural (nutritional behaviours, physical activity status and sleep patterns) of Turkish adult individuals during the COVID-19 pandemic period.

**Design:**

Cross-sectional online survey. The participants filled out a questionnaire (developed by using Google Forms) that contained descriptive characteristics, nutritional behaviours, sleep patterns, physical activity status, anthropometric measurements, COVID-19-related level of knowledge and the questions of the Center for Epidemiological Studies Depression Scale through e-mail or social media (WhatsApp).

**Setting:**

Turkey.

**Participants:**

Totally, 1120 adult individuals who completed an online survey between April and May 2020.

**Results:**

It was determined that 29·1 % of the individuals showed mild, 34·2 % moderate and 23·4 % severe depression symptoms during the pandemic period. A significant relationship was found between gender, age and educational status, marital status and depression levels of the individuals, respectively (χ^2^ = 35·292, χ^2^ = 103·46, χ^2^ = 24·524 and χ^2^ = 86·208, *P* < 0·05). The top three foods consumed most during the pandemic period are tea and coffee (66·6 %), pastry (e.g. cake and cookie) (56·4 %) and desserts (49·6 %). During the pandemic period, 42·5 % of the individuals stated that they slept more and 40·2 % stated that there was no change in their sleep patterns. Daily physical activity durations were determined as 8·25 ± 1·77 h for sleep, 4·21 ± 2·68 h for lying down, 5·42 ± 2·64 h for sitting and 6·16 ± 4·82 h for standing activities.

**Conclusion:**

It was determined that the individuals showed different levels of depression symptoms during the pandemic period. Especially, carbohydrate food consumption increased, and physical activity status and sleep patterns changed due to the increased time spent sitting and lying.

Coronavirus Disease 2019 has originated in Wuhan, China, spread all over the world between December 2019 and early 2020, and significantly threatened human health and life^(1,2)^. The WHO defined the new coronavirus as COVID-19 on 11 February 2020 and declared the situation pandemic on 11 March 2020^(3,4)^. Since the first case of COVID-19 of 11 March 2020, in Turkey, the total number of patients is 281 509, the rate of pneumonia seen in patients is 7·6 % and the total number of patients recovered is 252·152, while the total number of deaths is 6730^(5)^.

COVID-19 is a respiratory disease caused by the new coronavirus SARS-CoV-2, which has been declared pandemic^(3)^. The virus can be transmitted through the droplets spread from the infected individuals by coughing and sneezing, touching the contaminated surfaces and objects, and touching the mouth, nose or eyes with infected hands^(6)^. Its rapid spread, lack of treatment and lethal course make the effect of the virus important^(7)^. The most important way to prevent the disease is not to be exposed to the virus^(8)^.

There are some measures recommended during the pandemic period and implemented in our country to decrease the speed of the spread. Although the effects of the disease on children are weak, by taking into consideration the possibility of infection of adults by children, effective measures have been taken such as suspending schools, closing universities and dormitories, suspending meetings and events, starting working remotely, and declaring a short-term curfew in major cities for the individuals over the age of 65 years and below 18 years^(9)^.

In addition to the COVID-19 outbreak, incorrect information about the outbreak^(10)^, news on TV and social media^(11,12)^, travel restrictions and quarantines negatively affect people’s lives^(13)^. The pandemic period causes various psychological problems in individuals (such as panic, anxiety and depression)^(1,2,14,15)^, disrupted sleep patterns^(16,17)^ and physical immobilisation^(18,19)^. The boredom and stress cause an excessive intake of energy by consuming large amounts of fat, carbohydrates (sugary foods) and protein. These unhealthy nutritional behaviours increase the frequency of obesity and chronic diseases such as diabetes and heart disease, which increase COVID-19 complications^(20–23)^.

This epidemic is an extraordinary situation; it is very important to keep the immune system strong in terms of preventing infections, and protecting and maintaining health under extraordinary conditions^(24)^. The most effective methods include healthy and balanced nutrition, adequate physical activity and regular sleep^(25,26)^.

Scientific researches in Turkey is often COVID-19 studies to assess its impact on the emotions of individuals in the pandemic period^(27–29)^. No published scientific research has been found on the physical activity and nutritional status of individuals.

In this study, the aim was to evaluate emotional (depression) and behavioural (nutritional behaviours, physical activity status and sleep patterns) of Turkish adult individuals during the COVID-19 pandemic period.

## Methods

### Participants

The population of the research consisted of adults in Turkey. In the research, a sample selection method was not used. The individuals, who were between the ages of 18–65 years and agreed to participate voluntarily, were included in the research. Totally, 1120 individuals participated in the research.

### Study protocol

The research was carried out electronically by taking into account the current situation experienced due to the COVID-19 pandemic. The informed consent of the participants was obtained electronically before the questionnaire. The participants filled out a questionnaire (developed using Google Forms) that contained descriptive characteristics (age, gender, education, occupation, chronic disease status and marital status), nutritional behaviours (number of meals, food and supplement preferences, and appetite), sleep patterns (no change, I sleep more and I sleep less), physical activity status, anthropometric measurements (body weight and height), COVID-19-related level of knowledge and the questions of the Center for Epidemiological Studies Depression Scale (CES-D Scale) through e-mail or social media (WhatsApp).

The declarations of the participants were taken as a basis for body weight and height measurement values.

COVID-19 knowledge level was evaluated based on six basic questions addressed to the participants: (1) it is/can be transmitted by inhalation of droplets caused by infected/diseased persons through sneezing, coughing and speaking; (2) it is/can be transmitted by touching something that the infected/diseased person touches; (3) the incubation period of the virus does not exceed 14 d; (4) it is/can also be transmitted by contact with an asymptomatic (no signs of infection) person; (5) the disease can be treated with targeted drug studies; and (6) malaria drugs, beverages containing tonic water, drugs containing quinine are not protective against the disease. While each correct answer was evaluated as 1 point, the wrong answers were not scored. Regarding the COVID-19 disease, the participants with a score of ≥ 5 were evaluated as ‘Quite understand’, = 4 as ‘General understand’ and ≤ 3 as ‘Do not understand’^(30)^.

CES-D Scale is a four-point Likert-type scale (3 = agree, 2 = partially agree, 1 = disagree and 0 = no idea) consisting of 20 items in which depression symptoms are evaluated. The score that can be obtained from the scale varies between 0 and 60. Higher scores indicate more severe symptoms of depression. In the assessment, 0–9 points showed ‘Normal level’, 10–15 points showed ‘Mild depression’, 16–24 points showed ‘Moderate depression’ and 25–60 points showed ‘Severe depression’^(31–33)^.

### Statistical analysis

Descriptive analyses (percentage, mean and sd) were conducted to describe the demographic characteristic, the time of following developments related to coronavirus on television and the internet during the day, COVID-19-related knowledge, body weight, height, BMI, sleep, and physical activity status, nutritional status, and habits. The prevalence of gender, age, education, profession, marriage and chronic disease stratified by depression were reported, and the χ^2^ was used to compare the differences between groups. Multivariate logistic regression models were performed to explore potential influence factors (gender, age, education, profession, marriage and chronic disease) for depressive symptoms. Adjusted OR (AOR) and 95 % CI were obtained from logistic regression models. All data were analysed using Statistical Package for Social Sciences (SPSS) version 22.0. *P*-values of less than 0·05 were considered statistically significant.

## Results

A total of 1120 individuals, 412 (36·8 %) male and 708 (63·2 %) females, participated in the study. The mean age was determined as 33·04 ± 11·04. Among those who participated in the research, 84·6 % of individuals were university/bachelor’s/master’s degree graduates. As for profession, 37·4 % were teachers, 29·2 % were students and 10·6 % were self-employed. There was no chronic disease in 93·9 % of the individuals. The time of following developments related to coronavirus on television and the internet during the day was 0–1 h in 77·5 % of the individuals. In terms of the knowledge level of COVID-19, it was determined that 69·7 % of the individuals ‘quite understood’ the COVID-19 pandemic. The mean BMI of the individuals was calculated as 24·85 ± 9·71 kg/m^2^ (Table 1).

**Table 1:** Descriptive features of individuals (*n* 1120)

Features	*n*	%
Gender		
Male	412	36·8
Female	708	63·2
Age, year		
Mean	33·04	
sd	11·04	
Education status		
Primary school	17	1·5
Secondary school	22	2·0
High school	133	11·9
University/bachelor’s/master’s degree	948	84·6
Profession		
Officer	419	37·4
Worker	88	7·8
Self-employment	119	10·6
Retired	43	3·8
Housewife	91	8·1
Unemployed	35	3·1
Student	325	29·2
Marital status		
Married	576	51·4
Single	513	45·8
Widowed/divorced	31	2·8
Chronic disease status		
No	1052	93·9
Diabetes	19	1·7
Hypertension	31	2·8
CVD	18	1·6
Keeping up with the developments related to coronavirus on television and the internet during the day		
0–1 h	868	77·5
2–3 h	153	13·6
4–5 h	60	5·4
6 h or more	39	3·5
COVID-19 knowledge level		
Quite understand	781	69·7
General understand	252	22·5
Do not understand	87	7·8
Body weight, kg		
Mean	70·03	
sd	15·56	
Height, cm		
Mean	168·69	
sd	10·69	
BMI, kg/m^2^		
Mean	24·85	
sd	9·71	

The information regarding the sleep and physical activity status of the individuals are shown in Table 2. And 42·5 % of the individuals stated that they slept more during the pandemic period and 40·2 % stated that there was no change in their sleep patterns. Daily physical activity durations were determined as 8·25 ± 1·77 h for sleep, 4·21 ± 2·68 h for lying down, 5·42 ± 2·64 h for sitting and 6·16 ± 4·82 h for standing activities.

**Table 2:** Sleep and physical activity status of individuals (*n* 1120)

	*n*	%
Sleep patterns		
No change	450	40·2
I sleep more	476	42·5
I sleep less	194	17·3
Physical activity status		
Sleep (h)		
Mean	8·25	
sd	1·77	
Reaching out (rest and listening to music) (h)		
Mean	4·21	
sd	2·68	
Sitting jobs (at the computer, watching TV and reading a book) (h)		
Mean	5·42	
sd	2·64	
Light activities on foot (home cleaning, child care, laundry-dishwashing, cooking) (h)		
Mean	6·16	
sd	4·82	

The psychological status experienced by the individuals at different times during the pandemic period when compared to the same psychological status before the pandemic is given in Table 3.

**Table 3:** Individuals’ appetite according to different emotional status (*n* 1120)

	My appetite less than before the pandemic		My appetite is higher than before pandemic		My appetite is the same as before pandemic	
Emotional status	*n*	%	*n*	%	*n*	%
Stressful	347	31·0	392	35·0	381	34·0
Nervous	402	35·9	362	32·3	356	31·8
Happy	339	30·3	351	31·3	430	38·4
Sad	439	39·2	330	29·5	351	33·3
Excited	415	37·1	308	27·5	397	35·4
When you feel alone	395	35·3	357	31·9	363	32·9

The information regarding the individuals’ nutritional status and habits are shown in Table 4. The mean number of meals was 2·77 ± 1·04. In this period, while the body weight of 48·6 % of individuals did not change, it was found that body weight of 38·4 % of the participants increased. The most consumed foods during the pandemic period were determined as tea and coffee by 66·6 %, pastries by 56·4 % (e.g. pies, cakes and cookies) and desserts by 49·6 %. Due to the belief that they will strengthen their immune systems, the individuals mostly consumed fruits and vegetables (74·0 %), probiotics (e.g. yogurt, kefir) (55·0 %) and foods containing *n*-3 (36·2 %). The commonly used supplements were vitamin C (28·1 %), vitamin D (24·2 %), probiotics (18·2 %) and multivitamin-minerals (15·4 %). The consumption of supplements, such as Zn and *n*-3, was equal (11·1 % for both).

**Table 4:** Nutritional status and habits of individuals (*n* 1120)

	*n*	%
Number of meals		
Mean	2·77	
sd	1·04	
Change in body weight		
Increased	430	38·4
Decreased	146	13·0
Unchanged	544	48·6
The most consumed foods during the pandemic period*		
Sweet food	556	49·6
Ready snacks (biscuits, crackers and chips)	348	31·1
Nuts	457	40·8
Tea and coffee	747	66·6
Alcohol	40	3·5
Water	478	42·6
Vegetable and fruit	543	48·4
Pastries (pies, cakes and cookies)	632	56·4
Milk and yogurt	464	41·4
Food consumed by believing that it will strengthen immunity during the pandemic period*		
Vegetable and fruit	829	74·0
*n*-3 (Fish, walnut and purslane)	406	36·2
Probiotics (such as yogurt and kefir)	616	55·0
Dry beans	320	28·5
Supplement product consumed with the belief that it will strengthen immunity during the pandemic period*		
Probiotics	204	18·2
*n*-3	124	11·1
Vitamin D	271	24·2
C vitamin	315	28·1
Zn	124	11·1
Multivitamin-mineral	173	15·4

* Evaluation was made on more than one answer.

The relationship between the individuals’ gender, age, education status, profession, marital status, chronic disease status and depression status is shown in Table 5. And 13·3 % of individuals showed normal, 29·1 % mild, 34·2 % moderate and 23·4 % severe depression symptoms. The mean score of the CES-D Scale was determined as 19·02 ± 9·69. A significant relationship was found between gender, age and educational status, marital status and depression levels of the individuals, respectively (χ^2^ = 35·292, χ^2^ = 103·46, χ^2^ = 24·524 and χ^2^ = 86·208, *P* < 0·05).

**Table 5:** The relationship between individuals’ gender, age, education status, profession, marital status, chronic disease status and depression level (*n* 1120)

	Depression level											
	Normal		Mild depression		Moderate depression		Severe depression		Total			
	*n*	%	*n*	%	*n*	%	*n*	%	*n*	%	χ^2^	*P*
General distribution	149	13·3	326	29·1	383	34·2	262	23·4	1120	100·0		
Depression score												
Mean	19·02											
sd	9·69											
Gender												
Male	81	54·4	125	38·3	140	36·6	66	25·2	412	36·8	35·292	0·000*
Female	68	45·6	201	61·7	243	63·4	196	74·8	708	63·2		
Age, year												
< 35	47	31·5	142	43·6	232	60·6	200	76·3	621	55·4	103·460	0·000*
≥ 35	102	68·5	184	56·4	151	39·4	82	23·7	489	44·6		
Education status												
Primary school	1	0·7	11	3·4	4	1·1	1	0·4	16	1·4	24·524	0·017*
Secondary school	3	2·0	8	2·5	7	1·8	4	1·5	22	2·0		
High school	29	19·5	34	10·4	41	10·7	29	11·1	133	11·8		
University/bachelor’s/master’s degree	116	77·8	273	83·7	331	86·4	228	87·0	948	84·6		
Profession												
Officer	36	24·1	150	46·0	183	47·7	50	19·2	419	37·4		
Worker	21	14·1	43	13·1	15	3·9	9	3·2	88	7·8		
Self-employment	12	8·0	28	8·5	69	18·1	10	4·1	119	10·6		
Retired	12	8·0	10	3·0	9	2·3	12	4·5	43	3·8	18·754	0·080
Housewife	27	18·1	40	12·2	13	3·3	11	4·2	91	8·1		
Unemployed	2	1·3	3	1·0	5	1·3	25	9·5	35	3·1		
Student	39	26·4	52	16·2	89	23·4	145	55·3	325	29·2		
Marital status												
Married	103	69·1	210	66·4	176	46·0	87	33·2	576	51·0		
Single	43	28·9	104	31·9	197	51·4	169	64·5	513	45·8	86·208	0·000*
Widowed/divorced	3	2·0	12	3·7	10	2·6	6	2·3	31	2·8		
Chronic disease status												
No	138	92·6	301	92·3	359	93·7	254	96·9	1052	93·9		
Diabetes	2	1·3	6	1·8	9	2·3	2	0·8	19	1·7	15·764	0·072
Hypertension	4	2·7	14	4·3	12	3·1	1	0·4	31	2·8		
CVD	5	3·4	5	1·5	3	0·9	5	1·9	18	1·6		

* Pearson ki-kare test (*P* < 0·05).

**Table 6:** Multinominal logistic regression analysis results (*n* 1120)

	Depression level	
Variables	AOR	95 % CI
Gender		
Male	1·00	
Female	1·54	1·36, 1·80*
Age, year		
< 35	1·45	0·95, 2·21*
≥ 35	1·00	
Education status		
Primary school	1·00	
Secondary school	1·57	0·20, 1·71
High school	0·95	0·23, 2·38
University/bachelor’s/master’s degree	1·51	1·30, 1·88*
Profession		
Officer	1·00	
Worker	0·85	0·63, 1·80
Self-employment	1·05	0·84, 2·12
Retired	0·93	0·61, 1·98
Housewife	1·13	0·56, 2·05
Unemployed	1·22	0·76, 2·49
Student	0·92	0·58, 2·23
Marital status		
Married	1·00	
Single	1·53	1·15, 2·48*
Widowed/divorced	0·78	0·55, 1·10
Chronic disease status		
No	1·00	
Diabetes	0·65	0·39, 1·28
Hypertension	0·72	0·50, 1·49
CVD	0·98	0·65, 1·78

AOR, adjusted OR.

* *P* < 0·05.

According to the results of the multinominal logistic regression analysis, the female participants compared to the male participants (AOR = 1·54, 95 % CI: 1·36, 1·80), those under 35 years of age compared to the participants above 35 years of age (AOR = 1·45, 95 % CI: 0·95, 2·21), the participants with a university/bachelor’s/master’s degree as educational status (AOR = 1·51, 95 % CI: 1·30, 1·80) and single individuals (AOR = 1·53, 95 % CI: 1·15, 2·48) were associated with a higher risk of depression.

## Discussion

With the COVID-19 pandemic, the concern for the spread of the virus has necessitated infection control and safety measures. The implementation of ‘stay at home’ that has been initiated to restrict social life is a basic safety step that can limit infections^(18)^. Such a difference in social life causes some changes in individuals’ psychological status, physical activity levels and nutritional behaviours.

A total of 1120 individuals, 412 (36·8 %) male and 708 (63·2 %) female with a mean age of 33·04 ± 11·04, participated in this research that was conducted to determine the possible changes during the pandemic period. Of the patients, 84·6 % had a university/bachelor’s/master’s degree. When asked about following the developments regarding coronavirus on television and the internet during the day, 77·5 % of the individuals responded as 0–1 h, 13·6 % as 2–3 h, 5·4 % as 4–5 h and 3·5 % as 6 h and above. As a result of the answers given to the questions asked about COVID-19 knowledge levels, it was determined that 69·7 % of the participants understood the pandemic quite well, 22·5 % generally understood and 7·8 % did not understand. Huanga and Zhao (2020) determined that 78·8 % (5702) of 7236 participants were quite understood, 15·7 % (1136) generally understood % 5·5 (398) did not understand^(30)^.

It is stated that the psychological status or characteristics of individuals affect their eating behaviours^(33)^. Increased time spent at home, pandemic news listened and watched, increased anxiety, increased desire to consume food (especially carbohydrate foods) due to mood, and decreased physical activity can cause unwanted increases in body weight. Exercising routinely at home is important to increase the physical activity level of the body and maintain a healthy life during the pandemic period^(18)^. And 42·5 % of individuals stated that they slept more during the pandemic period and 40·2 % stated that there was no change in their sleep patterns. The mean daily activity duration was found as 8·25 ± 1·77 h for sleep, 4·21 ± 2·68 h for works performed lying down, 5·42 ± 2·64 h for works performed sitting and 6·16 ± 4·82 h for light standing activities. As a result, it was determined that most of the day was spent sitting and lying down. The mean BMI was 24·85 ± 9·71 kg/m^2,^ and it was determined that the body weight did not change in 48·6 % of the individuals during the pandemic period, while the body weight increased by 38·4 % of them.

In addition to physically affecting individuals, COVID-19 increased susceptibility to psychological problems such as depression, anxiety and panic disorder^(30,34)^. In this study, the mean score received from the CES-D Scale was determined as 19·02 ± 9·69. Of the participants, 13·3 % show normal, 29·1 % mild, 34·2 % moderate and 23·4 % severe depression symptoms. A significant correlation was found between the individuals’ gender, age and educational status and their depression levels (χ^2^ = 35·292, χ^2^ = 103·46 and χ^2^ = 24·524, *P* < 0·05). According to the results of the multinominal logistic regression analysis, the female participants compared to the male participants (AOR = 1·54, 95 % CI: 1·36, 1·80), those under 35 years of age compared to the participants above 35 years of age (AOR = 1·45, 95 % CI: 0·95, 2·21) and the participants with university/bachelor’s/master’s degree as educational status (AOR = 1·51, 95 % CI: 1·30, 1·80) were associated with a higher risk of depression. In their study, Huanga and Zhao (2020) found similar results; they determined that the risk of depression was higher in individuals under the age of 35 years compared to the individuals above 35 years (AOR = 1·77, 95 % CI: 1·58, 2·07)^(30)^. The pandemic situation in China caused an increase in depression, anxiety and panic attacks in humans^(8)^. Ahmed et al. (2020) found that 37·1 % of the participants (*n* 1074) had depression (mild 10·2 %, moderate 17·8 % and severe 9·1 %), and this rate was higher in the 21–30 years of age group^(35)^. In the study conducted on 7236 people, it was determined that there was a general anxiety state (35·1 %), depression (20·1 %) and a decrease in sleep quality (18·2 %) in the individuals during the pandemic period^(30)^. Anxiety and depression were more common among young people (< 35 years) and people who spent a lot of time on the internet and TV by reading and watching the news about COVID-19 (≥ 3 h/d)^(30)^. Since young people spend more time outside in their normal lives, having to stay at home caused more psychological problems.

The increase in the time spent at home changed the psychological status of the individuals but also caused them to receive more energy in their diets and consume more amounts of fat, carbohydrates and protein^(20)^. In a study investigating the relationship between negative psychological status and high food intake, it was found that negative mood increased food intake more than positive mood^(36,37)^. When the individuals’ appetite status during the pandemic period were compared to their appetite status before the pandemic period, it was found that when they were more stressful (35·0 %), they ate more, when they felt less nervous (35·9 %), sad (39·2 %), excited (37·1 %) and lonely (35·3 %), they ate less, and when they were happy, their appetite status did not change (38·4 %).

Stress causes individuals to search for food that will provide relief quickly, and generally, the tendency towards sugary foods increases^(38,39)^. The most consumed foods during the pandemic period were determined as tea and coffee by 66·6 %, pastries by 56·4 % (pies, cakes and cookies) and desserts by 49·6 %. In terms of food craving, it is stated that there is a gender difference, and it is remarkably higher in females compared to males^(38)^. The results of our study support this information.

There are insufficient data from clinical studies on the effects of nutrients on COVID-19 and the effects of nutritional supplements on COVID-19 prevention in healthy individuals. Although there is no definitive evidence due to the limitation of the studies, it is stated that balanced diets including probiotic-containing foods, immune-enhancing micronutrients such as polyphenols, vitamins A, C, E, D, B_1_, B_6_ and B_12_, and some minerals (especially Se, Zn, and Fe) may be effective in supporting immunity against COVID-19^(40–45)^. However, most guidelines emphasise the importance of fruits, vegetables, whole-grain foods, and vitamins A, C, D and Zn in protecting the immune system^(46)^. While it has been stated that supplementation of vitamin C and D, Zn, and Se as supplements may be beneficial for people with respiratory tract viral infections or those at risk and those with nutritional deficiencies, scientific research is limited and the data are not clear^(47,48)^. Also, there are findings that *n*-3 PUFA can benefit both the physiological and psychological effects of COVID-19 and support immunity^(48,49)^. In this study, believing that it will support the immune system, the most consumed foods are fruits and vegetables (74·0 %) and probiotics (55·0 %). The commonly used supplements were vitamin C (28·1 %), vitamin D (24·2 %), probiotics (18·2 %) and multivitamin-minerals (15·4 %). Those who preferred Zn and *n*-3 as supplements were equal (11·1 % for both). As a result of this study, it has been determined that individuals increase their consumption of vegetables and fruits and probiotics as food and vitamin C as a supplement with the belief of supporting immunity and protecting from COVID-19.

This study has some limitations. First, it is difficult to make causal inferences because the data and related analyses presented here were derived from a cross-sectional design. Second, the study was limited to the COVID-19 outbreak, and we used a web-based survey method to avoid possible infections. That causes the sampling of our study was voluntary and to be conducted online. Therefore, the possibility of selection bias should be considered. Finally, we were unable to assess an individual’s psychological state before the outbreak due to the sudden occurrence of the disaster.

As a result, it was determined that the pandemic period caused changes in the nutrition, physical activity and psychological status of the individuals participating in the research. It was determined that the individuals had different levels of depression risk, and the highest risk was observed in the females under 35 years of age and those with higher educational levels. It was determined that the consumption of carbohydrates increased, physical activity levels decreased and sleep patterns changed due to the increase in the time spent sitting and lying down.

## References

[1] Jakovljevic M , Bjedov S , Jaksic N et al. (2020) COVID-19 pandemia and public and global mental health from the perspective of global health security. Psychiatr Danub 32, 6–14. 32303023 10.24869/psyd.2020.6

[2] Qiu J , Shen B , Zhao M et al. (2020) A nationwide survey of psychological distress among Chinese people in the COVID-19 epidemic: implications and policy recommendations. Gen Psychiatr 33, e100213. 32215365 10.1136/gpsych-2020-100213 PMC7061893

[3] World Health Organization (2020) Director-General’s remarks at the media briefing on 2019-nCoV on February 11, 2020. https://www.who.int/dg/speeches/detail/who-director-general-s-remarks-at-the-media-briefing-on-2019-ncov-on-11-february-2020 (accessed February 2020).

[4] WHO (2020) WHO Director-General’s Opening Remarks at the Media Brieﬁngon COVID-19-March 2020. https://www.who.int/dg/speeches/detail/who-director-general-s-opening-remarks-at-the-media-brieﬁng-on-covid-19—11-march-2020 (accessed March 2020).

[5] Republic of Turkey. Ministry of Health. https://covid19bilgi.saglik.gov.tr/tr/ (accessed September 2020).

[6] Republic of Turkey. Ministry of Health and General Directorate of Public Health (2020) COVID-19 Quide. https://hsgm.saglik.gov.tr/tr/bulasici-hastaliklar/2019-n-cov.html (accessed March 2020).

[7] Aslan MM , Yuvacı UH , Köse O et al. (2010) COVID-19 and pregnancy. J Biotechnol Strateg Health Res 1, 10–13.

[8] Centers for Disease Control and Prevention (2020) Coronavirus Disease 2019 (COVID-19) How to Protect Yourself. https://www.cdc.gov/coronavirus/2019-ncov/prepare/prevention.html (accessed March 2020).

[9] European Centre for Disease Prevention and Control (2020) Novel coronavirus disease 2019 (COVID-19) pandemic: increased transmission in the EU/EEA and the UK – sixth update. ECDC. https://www.ecdc.europa.eu/sites/default/files/documents/RRA-sixth-update-Outbreak-ofnovel-coronavirus-disease-2019-COVID-19.pdf (accessed March 2020).

[10] Shimizu K (2020) 2019-nCoV, fake news, and racism. Lancet 395, 685–686. 10.1016/S0140-6736(20)30357-3 PMC7133552 32059801

[11] Gao J , Zheng P , Jia Y et al. (2020) Mental health problems and social media exposure during COVID-19 outbreak. PLoS One 15, e0231924. 32298385 10.1371/journal.pone.0231924 PMC7162477

[12] Goyal K , Chauhan P , Chhikara K et al. (2020) Fear of COVID 2019: first suicidal case in India. Asian J Psychiatr 49, 101989. 32143142 10.1016/j.ajp.2020.101989 PMC7130010

[13] Nguyen HC , Nguyen MH , Do BN et al. (2020) People with suspected COVID-19 symptoms were more likely depressed and had lower health-related quality of life: the potential benefit of health literacy. J Clin Med 9, E965. 32244415 10.3390/jcm9040965 PMC7231234

[14] Wang J , Wang JX & Yang GS (2020) The psychological impact of COVID-19 on Chinese individuals. Yonsei Med J 61, 438–440. 32390368 10.3349/ymj.2020.61.5.438 PMC7214113

[15] Roy D , Tripathya S , Kar SK et al. (2020) Study of knowledge, attitude, anxiety & perceived mental healthcare need in Indian population during COVID-19 pandemic. Asian J Psychiatry 51, 1–7. 10.1016/j.ajp.2020.102083 PMC7139237 32283510

[16] Bao Y , Sun Y , Meng S et al. (2020) 2019-nCoV epidemic: address mental health care to empower society. Lancet 395, e37–e38. 32043982 10.1016/S0140-6736(20)30309-3 PMC7133594

[17] Altena E , Baglioni C , Espie CA et al. (2020) Dealing with sleep problems during home confinement due to the COVID-19 outbreak: practical recommendations from a task force of the European CBT-I Academy. Sleep Res 4, e13052. 10.1111/jsr.13052 32246787

[18] Chen P , Mao L , Nassis GP et al. (2020) Coronavirus disease (COVID-19): the need to maintain regular physical activity while taking precautions. J Sport Health Sci 9, 103–104. 32099716 10.1016/j.jshs.2020.02.001 PMC7031771

[19] Ceravolo M , De Sire A , Andrenelli E et al. (2020) Systematic rapid “living” review on rehabilitation needs due to covid-19: update to March 31, 2020. Eur J Phys Rehabil Med 56, 347–353. 32316718 10.23736/S1973-9087.20.06329-7

[20] Moynihan AB , Van Tilburg WA , Igou ER et al. (2015) Eaten up by boredom: consuming food to escape awareness of the bored self. Front Psychol 6, 369. 25883579 10.3389/fpsyg.2015.00369 PMC4381486

[21] Yılmaz C & Gökmen V (2020) Neuroactive compounds in foods: occurrence, mechanism, and potential health effects. Food Res Int 128, 108744. 31955786 10.1016/j.foodres.2019.108744

[22] Wu C , Chen X , Cai Y et al. (2020) Risk factors associated with acute respiratory distress syndrome and death in patients with coronavirus disease 2019 pneumonia in Wuhan, China. JAMA Intern Med 13, e200994. 10.1001/jamainternmed.2020.0994 PMC7070509 32167524

[23] Butler MJ & Barrientos RM (2020) The impact of nutrition on COVID-19 susceptibility and long-term consequences. Brain, Behav, Immun 87, 53–54. 32311498 10.1016/j.bbi.2020.04.040 PMC7165103

[24] Chowdhury MA , Hossain N , Kashem MA , et al. (2020) Immune response in COVID-19: a review. J Infect Public Health. Published online: 14 July 2020. doi: 10.1016/j.jiph.2020.07.001. PMC7359800 32718895

[25] FAO (2020) Maintaining a healthy diet during the COVID-19 pandemic. 10.4060/ca8380en (accessed April 2020).

[26] Khaled MB & Benajiba N (2020) The role of nutrition in strengthening immune system against newly emerging viral diseases: case of SARS-CoV-2. North Afr J Food Nutr Res 4, 240–244.

[27] Özdin S & Özdin ŞB (2020) Levels and predictors of anxiety, depression and health anxiety during COVID-19 pandemic in Turkish society: the importance of gender. Int J Soc Psychiatr 66, 504–511. 10.1177/0020764020927051 PMC7405629 32380879

[28] Ustun G (2020) Determining depression and related factors in a society affected by COVID-19 pandemic. Int J Soc Psychiatr 30, 20764020938807. 10.1177/0020764020938807 PMC7331110 32605422

[29] Seçer İ & Ulaş S (2020) An investigation of the effect of COVID-19 on OCD in youth in the context of emotional reactivity, experiential avoidance, depression and anxiety. Int J Ment Health Addict 13, 1–14. 10.1007/s11469-020-00322-z PMC7293436 32837429

[30] Huang Y & Zhao N (2020) Generalized anxiety disorder, depressive symptoms and sleep quality during COVID-19 outbreak in China: a web-based cross-sectional survey. Psychiatr Res 288, 112954. 10.1016/j.psychres.2020.112954 PMC7152913 32325383

[31] Tatar A & Saltukoglu G (2010) The adaptation of the CES-Depression Scale into Turkish through the use of confirmatory factor analysis and item response theory and the examination of psychometric characteristics. Bull Clin Psychopharmacol 20, 213–227.

[32] Weissman MM , Sholomskas D , Pottenger M et al. (1977) Assessing depressive symptoms in five psychiatric populations: a validation study. Am J Epidemiol 106, 203–214. 900119 10.1093/oxfordjournals.aje.a112455

[33] Moon JR , Huh J , Song J et al. (2017) The center for epidemiologic studies depression scale is an adequate screening instrument for depression and anxiety disorder in adults with congenital heart disease. Health Qual Life Outcomes 15, 176. 28874154 10.1186/s12955-017-0747-0 PMC5585982

[34] Hiremath P , Kowshik S & Manjunath M (2020) COVID 19: impact of lock-down on mental health and tips to overcome. Asian J Psychiatr 51, 102088. 32302964 10.1016/j.ajp.2020.102088 PMC7151434

[35] Ahmed Md Z , Ahmed O & Aibao Z (2020) Epidemic of COVID-19 in China and associated psychological problems. Asian J Psychiatr 51, 102092. 32315963 10.1016/j.ajp.2020.102092 PMC7194662

[36] Serin Y & Şanlıer N (2018) Emotional eating factors affecting intake and basic nursing approaches. J Psychiatric Nurs 9, 135–146.

[37] Evers C , Adriaanse M , de Ridder DT et al. (2013) Good Mood Food. Positive emotion as a neglected trigger for food ıntake. Appetite 68, 1–7. 23602962 10.1016/j.appet.2013.04.007

[38] Muscogiuri G , Barrea L , Savastano S et al. (2020) Nutritional recommendations for CoVID-19 quarantine. Eur J Clin Nutr 1–2. 10.1038/s41430-020-0635-2 PMC7155155 32286533

[39] Özenoğlu A (2018) Relationship between mood, food and nutrition. ACU Sağlık Bil Derg 9, 357–365.

[40] Olaimat AN , Aolymat I , Al-Holy M et al. (2020) The potential application of probiotics and prebiotics for the prevention and treatment of COVID-19. NPJ Sci Food 4, 1–7. 10.1038/s41538-020-00078-9 PMC7536434 33083549

[41] Lange KW & Nakamura Y (2020) Food bioactives, micronutrients, immune function and COVID-19. J Food Bioact 10, 1–8.

[42] Eskici G (2020) COVID-19 pandemia: nutrition recommendations for quarantine. Anatol Clin 25, 124–129.

[43] Richardson DP (2020) Making nutrition a priority to help reduce risk of infections and death during the coronavirus pandemic. BMJ 369, m1327.32238354

[44] Calder PC , Carr AC , Gombert AF et al. (2020) Optimal nutrition status for a well-functioning immune system is an important factor to protect against viral infections. Nutrients 12, E1181. 32340216 10.3390/nu12041181 PMC7230749

[45] Lanham-New S , Webb AR , Cashman KD et al. (2020) Vitamin D and SARS-CoV-2 virus/COVID-19 disease. BMJ Nutr Prev Health 3, e000089. 10.1136/bmjnph-2020-000089 PMC7246103 33230499

[46] de Faria Coelho-Ravagnani C , Corgosinho FC , Sanches FLFZ et al. (2020) Dietary recommendations during the COVID-19 pandemic. Nutr Rev. Published online: 12 July 2020. doi 10.1093/nutrit/nuaa067. PMC7454801 32653930

[47] Chandra RK (1992) Effect of vitamin and trace-element supplementation on immune responses and infection in elderly subjects. Lancet 340, 1124–1127. 1359211 10.1016/0140-6736(92)93151-c

[48] Chang JPC , Pariantec CM & Su KP (2020) *n*-3 fatty acids in the psychological and physiological resilience against COVID-19. Prostaglandins Leukot Essent Fatty Acids 25, 102177. 10.1016/j.plefa.2020.102177 PMC7516470 33031994

[49] Khoramipour K , Basereh A , Hekmatikar AA et al. (2020) Physical activity and nutrition guidelines to help with the fight against COVID-19. J Sports Sci 25, 1–7. 10.1080/02640414.2020.1807089 32842905

